# Novel gel‐immersion endoscopic injection sclerotherapy method for prophylactic hemostasis of esophageal varices: A pilot feasibility and safety study (with video)

**DOI:** 10.1002/deo2.70056

**Published:** 2025-01-13

**Authors:** Noriaki Sugawara, Taro Iwatsubo, Yosuke Mori, Kazuki Takayama, Shun Sasaki, Noriyuki Nakajima, Hironori Tanaka, Akitoshi Hakoda, Satoshi Harada, Kazuhiro Ota, Toshihisa Takeuchi, Hiroki Nishikawa

**Affiliations:** ^1^ Second Department of Internal Medicine Osaka Medical and Pharmaceutical University Osaka Japan; ^2^ Endoscopy Center Osaka Medical and Pharmaceutical University Hospital Osaka Japan

**Keywords:** digestive system endoscopy, endoscopic injection sclerotherapy, esophageal varices, gel immersion endoscopy, prophylactic hemostasis

## Abstract

Endoscopic injection sclerotherapy (EIS) is a useful prophylactic hemostatic procedure for esophageal varices. However, injecting sclerosing agents into blood vessels is technically challenging and often ineffective. Gel‐immersion EIS (GI‐EIS) may facilitate easier intravascular sclerosing agent injection by dilating the varices and enhancing scope stability by maintaining low intra‐gastrointestinal pressure. Therefore, we aimed to evaluate the effectiveness and safety of this procedure. This retrospective study included 18 patients (14 men and four women; median age, 70 years; age range, 18–83 years) who underwent GI‐EIS at Osaka Medical Pharmaceutical University Hospital between December 1, 2022, and January 30, 2024. Patients who were at least 18 years of age at the time of treatment were included. No patients were excluded from the study. Thirty‐four punctures were performed. The donor vessel angiography success rate was 88.2% (30 of 34 punctures). The clinical success rate was 94.4% (17 of 18 patients). Esophageal varices in most patients disappeared or were reduced by 1 month after treatment. Adverse events related to the procedure included fever (three patients) and chest pain (one patient); however, both were resolved with conservative treatment. No respiratory deterioration due to aspiration occurred during the procedure. The results of this study demonstrate that GI‐EIS is a safe, clinically feasible, and effective treatment option for prophylactic hemostasis of esophageal varices.

## INTRODUCTION

Esophageal varices caused by portal hypertension, such as those that occur with cirrhosis of the liver,^1^ can rupture and lead to gastrointestinal bleeding and even death. Therefore, appropriate prophylactic hemostasis is crucial. Endoscopic injection sclerotherapy (EIS) and endoscopic variceal ligation (EVL) are common and effective prophylactic hemostatic procedures for the management of esophageal varices.^2^, ^3^ Although effective, EIS is a relatively challenging procedure due to difficulties associated with puncturing the varicose vein. Specifically, body movements and respiratory fluctuations can dislodge the needle or cause penetration of the vessel during insertion in a varicose vein and injection of sclerosing agents, resulting in bleeding. In contrast, gel‐immersion endoscopy, which has been applied to several endoscopic procedures such as endoscopic hemostasis and endoscopic mucosal resection,^4^, ^5^ facilitates a clear view and reduces internal pressure in the digestive tract, thereby improving scope stability.^6^ We previously developed an EIS method comprising gel‐immersion endoscopy.^7^ However, the usefulness of gel‐immersion EIS (GI‐EIS) is poorly understood; therefore, this study aimed to evaluate the efficacy and safety of GI‐EIS.

## PROCEDURE/TECHNIQUE

### Study design

This retrospective study included 18 patients (14 men and four women; median age, 70 years; age range, 18–83 years) who underwent GI‐EIS at Osaka Medical Pharmaceutical University Hospital between December 1, 2022, and January 30, 2024. The inclusion criteria were as follows: 18 years of age or older at the time of treatment and the presence of F2 or F3 esophageal varices or varices with the red color (RC) sign. The patients enrolled in this study were consecutive cases treated during the study period. No patients were excluded.

The severity of liver cirrhosis was assessed using the Child–Pugh classification system, which is a widely accepted tool used to evaluate liver function. This classification stratifies patients into three categories (A, B, and C) based on a scoring system that incorporates the following five clinical and biochemical parameters: bilirubin, albumin, prothrombin time, ascites, and hepatic encephalopathy. Patients with lower scores are categorized as class A, indicating well‐compensated cirrhosis, whereas patients with higher scores are categorized as class B or class C, which reflect progressively worse liver function and prognosis. Adverse events associated with GI‐EIS procedures were evaluated according to the severity grading system of the American Society for Gastrointestinal Endoscopy.^8^

The study protocol was approved by the Human Research Committee of Osaka Medical and Pharmaceutical University (Institutional Review Board No. 2024‐024) and conformed to the ethical guidelines of the 1975 Declaration of Helsinki.

### GI‐EIS procedure

**FIGURE 1:**
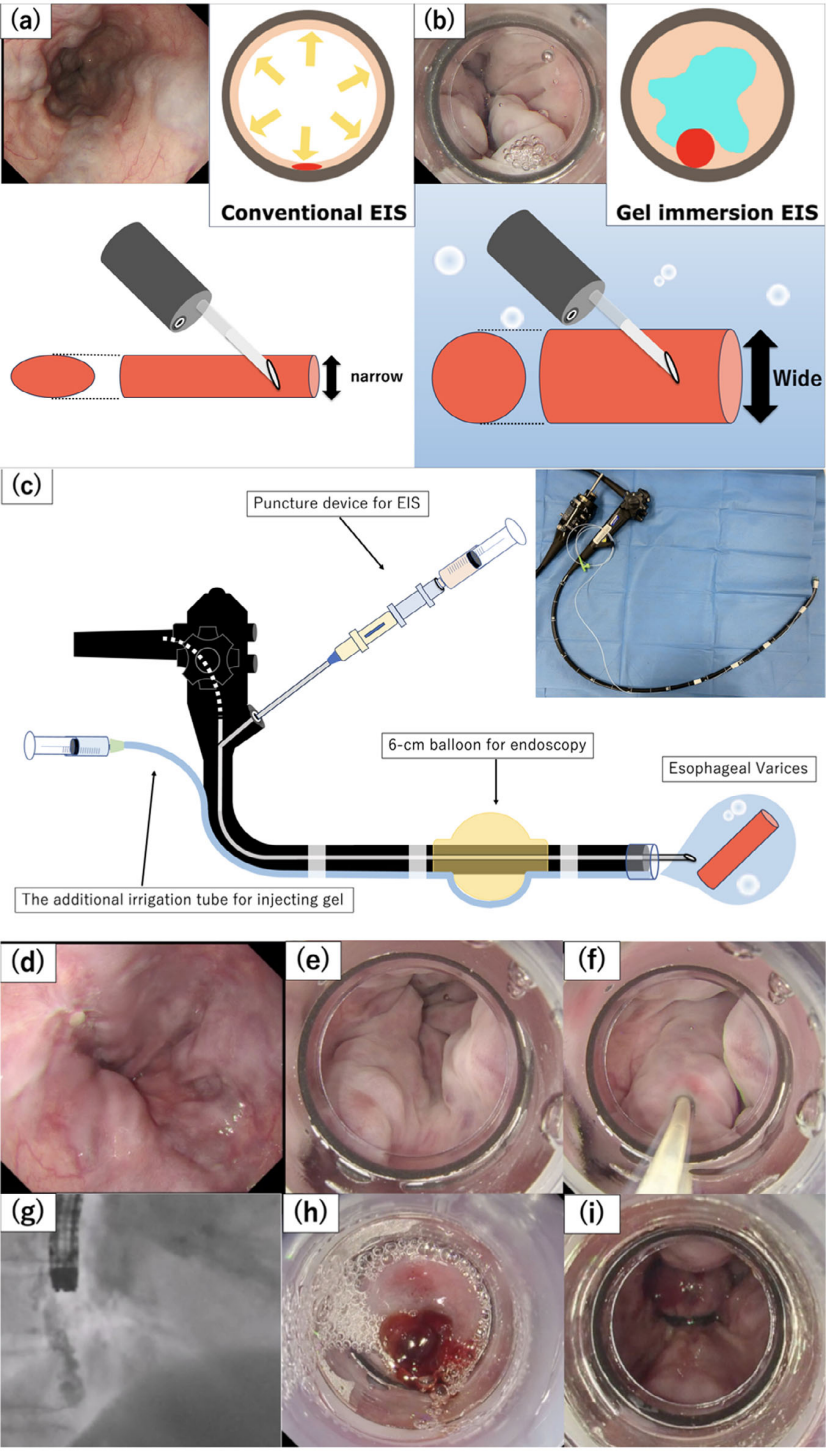
Schemas and typical intra‐procedure images of gel‐immersion endoscopic injection sclerotherapy (GI‐EIS) and those of conventional EIS for comparison. (a, b) Schema comparing the conventional EIS method with the GI‐EIS method. With the GI‐EIS method, the varicose vein is dilated and the lumen is wider, thus allowing easier puncture. (c) Schema and image of the GI‐EIS method. GI‐EIS is performed using an external irrigation tube (ERCP‐Catheter; MTW Endoskopie W Haag KG) for additional gel infusion. (d) Multiple F2 esophageal varices are present in the lower esophagus. (e) After the air is deflated from the esophagus and stomach, a sufficient amount of gel is injected to fill the lumen of the esophagus. With the gel, the pressure in the esophageal lumen is reduced and the varices are dilated. (f) After puncturing the varicose vein and confirming the presence of backflow, a sclerosing agent is injected. (g) A sufficient amount of sclerosis agent is injected to confirm that the donor vessel has achieved contrast under fluoroscopy. (h and i) After injecting a sufficient amount of sclerosing agent, the needle is removed and the puncture site is ligated.

GI‐EIS was performed as detailed in Video S1. Technical illustrations and images of GI‐EIS are shown in Figure 1. All procedures were performed by two board‐certified endoscopists (Noriaki Sugawara and Yosuke Mori) who are members of the Japan Gastroenterological Endoscopy Society. Prior to the procedure, the varicose vein was evaluated and cleaned using conventional endoscopic observation under insufflation. Then, an overtube (double type; TOP Corporation) was inserted, and the scope was removed. After attaching the modified pneumo‐activated EVL device (Sumitomo Bakelite Co. Ltd.) and a 6‐cm balloon for endoscopy (size: φ11 × 60 mm; CREATE MEDIC Co. Ltd.) to the tip of the scope, the scope was reinserted. After aspirating and degassing the air in the esophagus and stomach, the attached balloon was inflated to inhibit the varicose vein annulus. Subsequently, the gel was injected through the forceps channel to fill the esophageal lumen. When a sufficient amount of gel had accumulated, a puncture needle for EIS was inserted through the forceps channels. Then, the varicose vein was punctured under the gel. After confirming blood backflow, a contrast agent was injected to ensure proper contrast of the vessel. Next, the sclerosing agent, which comprised a mixture of 10% ethanolamine oleate (EO) and a non‐ionic angiographic contrast agent (iopamidole) diluted to a 5% concentration, was injected in the blood vessel using a 23‐gauge disposable puncture needle (Variceser type S; TOP Corporation).

After injecting a sufficient amount of sclerosing agent and confirming occlusion of the donor vein, the puncture needle was removed. The bleeding from the puncture site was stopped using balloon compression or the EVL technique.

### Post‐treatment follow‐up algorithm

Endoscopy was performed to determine efficacy at 1 week after GI‐EIS. If the effect was inadequate, then additional EVL was performed, and endoscopy was repeated 1 week later to determine the effectiveness of EVL. If the endoscopic examination confirmed satisfactory results, then the patient was discharged the following day. Follow‐up endoscopic examinations were performed at 1 month, 6 months, and 1 year after discharge.

This study included one patient whose observation period was less than 180 days; furthermore, this patient did not undergo endoscopic evaluation at 1 year following the procedure. However, this patient experienced an early recurrence of varices due to hepatocellular carcinoma progression; therefore, repeat treatment was performed. We included this patient in our study because clinical success was not achieved and we considered this case important to the evaluation of the efficacy of this technique.

### Definition

Evaluations of esophageal varices should adhere to the general rules of the Japan Society for Portal Hypertension for recording the endoscopic findings of esophageal and gastric varices.^9^ In this study, the technical success of EIS was defined as puncturing the varicose vein and injecting sufficient EO to confirm that the donor's vessel exhibited contrast under fluoroscopy. The clinical success of EIS was defined as the reduction or disappearance of esophageal varices following the procedure. The clinical outcome of EIS was classified into the following four categories according to the results of the endoscopic evaluation 1 month after the procedure: “disappearance”, indicating a decrease in the varicose vein form and the absence of high‐risk bleeding indicators, such as the RC sign; “reduction”, indicating a decrease in the varicose vein size without complete disappearance of the RC sign; “no change”, indicating the absence of differences after treatment; and “progressive worsening”, indicating worsening of varicose veins or the presence of the RC sign. The clinical success of EIS was defined as the reduction or disappearance of esophageal varices. Additionally, variceal recurrence referred to the occurrence of esophageal varices that required retreatment during the observation period.

### Endoscopy equipment and devices related to GI‐EIS

All GI‐EIS procedures were performed using a GIF‐H290T endoscope and an EVIS‐X1 endoscopic system (Olympus Optical Co., Ltd.). VISCOCLEAR (Otsuka Pharmaceutical Factory) was used as the gel product for GI‐EIS. When required, additional gel was added using an external irrigation tube during the procedure (ERCP‐Catheter; MTW Endoskopie W Haag KG). Conversely, saline solution was provided through a secondary water delivery tube with a water jet function. In other words, gel and water could be added independently through the additional tube and endoscope equipped with the water jet function, as necessary.

### Technique tips and troubleshooting

**FIGURE 2:**
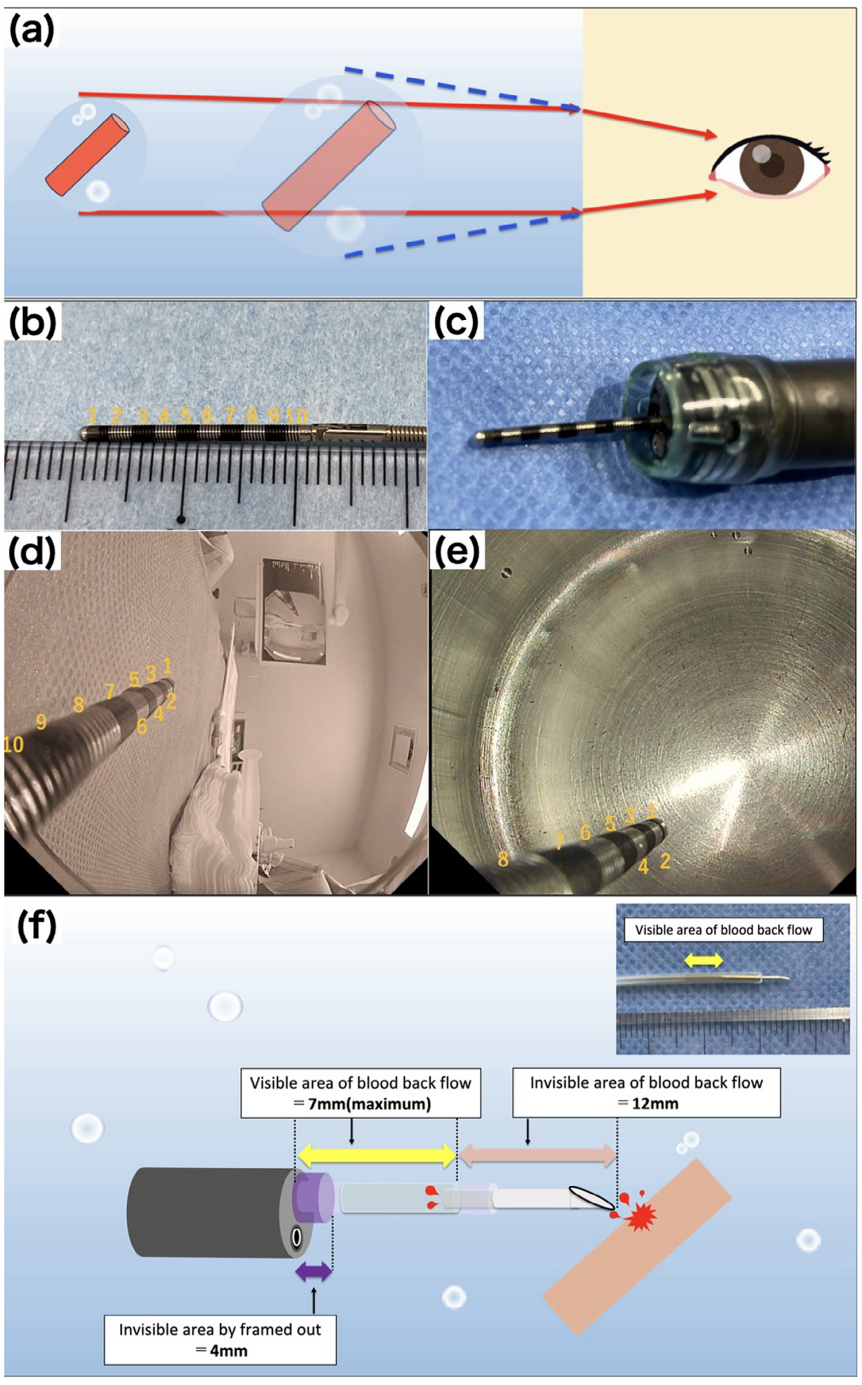
(a) With the gel, the endoscopic image is magnified and appears closer because of the difference in the refractive index. (b) An image of a measuring tool used for endoscopes, with 1 scale point comprising 2 mm (A, bendy type; Olympus Optical Co., Ltd.). (c) The measuring tool is fixed at the tip of the scope and actual endoscopic images obtained with gel as well as with air are shown. (d) Image of air‐immersion endoscopy. Almost all measurements at the scope tip are visible on the endoscopic image. (e) Image of gel‐immersion endoscopy. Approximately 4 mm of the measurement is outside the endoscopic field of view in the foreground and is not visible.

In contrast to the conventional EIS method, GI‐EIS often involves unique problems because the procedure is performed using gel. Schemas and images of these problems and countermeasures caused by the differences in refractive indices are shown in Figure 2. With the gel‐immersion technique, similar to the underwater technique, the endoscopic image is magnified and appears closer due to the difference in the refractive index. Consequently, the area that requires visualization to evaluate backflow after puncture may be out of frame or invisible. To prevent this, a certain margin between the tip of the scope and the puncture site is necessary. In our study, a puncture site 16 mm from the tip of the scope allowed the endoscopic image to include the site where the reverse hemorrhage was confirmed. When the pneumo‐activated EVL device (Sumitomo Bakelite Co., Ltd.) was attached to the endoscope tip, the distance from the forceps channel to the device tip was approximately 15 mm; we used this point as a marker for puncture procedures.

**FIGURE 3:**
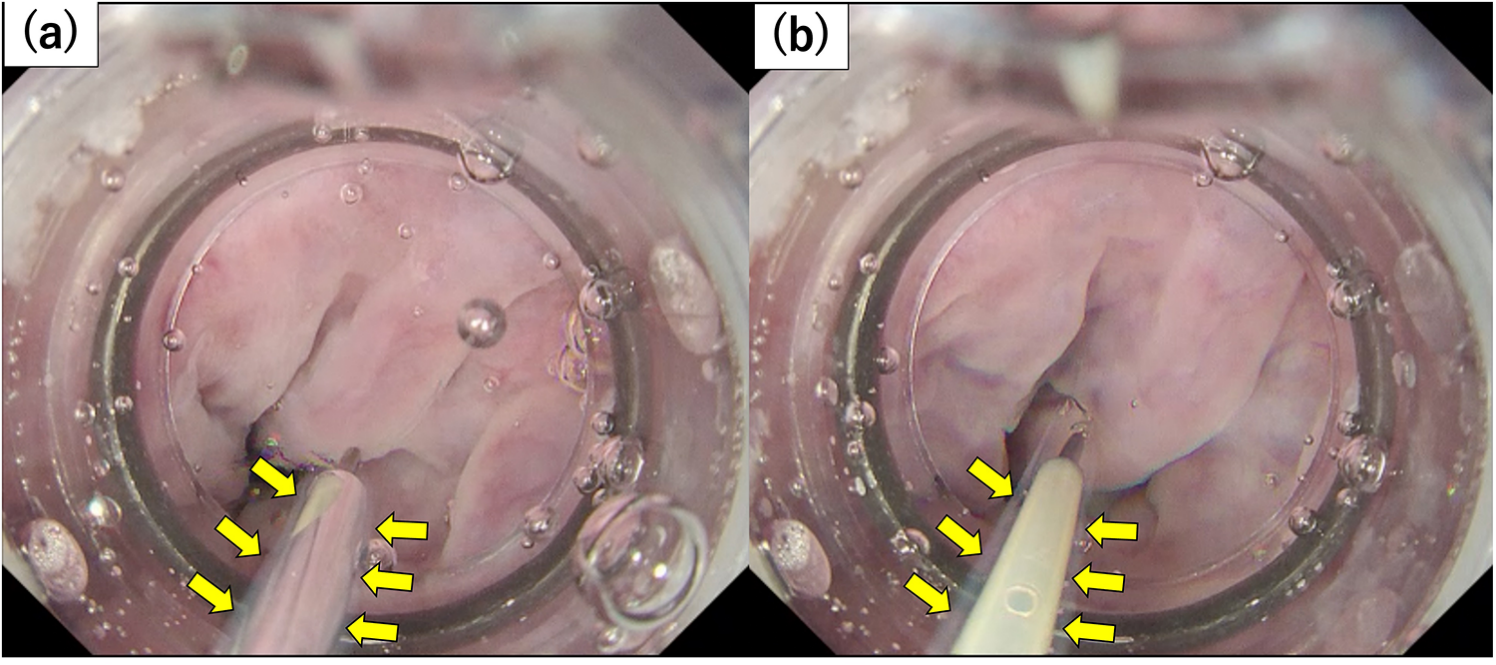
Images of the total reflections. (a) Total reflection has occurred, and the lumen of the puncture needle is reflected and invisible, similar to a mirror surface. (b) Image obtained after the lumen was filled with saline solution and air was flushed out. The lumen of the puncture needle is clearly visible.

To prevent total reflection, the lumen of the needle or sheath should be filled with saline solution mixed with an angiographic contrast agent, and air should be flushed from the sheath before puncturing the varicose vein. These procedures help reduce the refractive index difference, allow clear visualization of the needle lumen, prevent blood coagulation, and address the issue of air remaining in the lumen of the puncture needle. Care is necessary when performing multiple punctures during one session because air can enter the needle and cause total reflection. Air in the lumen causes total reflection in the outer tube of the needle due to the refractive index difference, thus obscuring the lumen of the needle and making it impossible to confirm the backflow of the blood. Representative endoscopic images obtained when total reflection occurred are shown in Figure 3.

### Statistical analysis

Descriptive statistics are presented as the mean and standard deviation or the median and range for continuous variables and as the number and frequency for categorical variables. All data were statistically analyzed using JMP Pro 17.2.0 software (SAS Institute).

## RESULTS

Table 1 presents patient characteristics and GI‐EIS outcomes. The severity of cirrhosis was classified according to the Child–Pugh criteria. Thirteen patients (72.2%) were classified as grade A and five patients (27.8%) were classified as grade B. We did not encounter any patients classified as grade C. During the procedure, the median number of varicose vein punctures was 2.0 (range, 1–4 punctures). Overall, the total number of punctures was 34, and 30 (88.2%) successful donor vessel angiographies were performed. For two of the four cases involving unsuccessful punctures, the backflow of blood could not be identified due to the total reflex phenomenon. For the remaining two cases, puncture of the thin F1 varix resulted in vessel penetration and subsequent bleeding. The clinical success rate was 94.6% (17/18). Treatment‐related adverse events included fever in three patients (16.6%) and chest pain in two patients (11.1%). These adverse events were transient and resolved spontaneously without specific treatment. However, none of the patients experienced adverse events such as pneumonia, vascular embolism, or esophageal stricture.

**TABLE 1:** Patient characteristics and treatment outcomes of the gel‐immersion endoscopic injection sclerotherapy (GI‐EIS) method.

	GI‐EIS
	*N* = 18
Age, mean (SD), years	70.00 (15.02)
Sex, *n* (%)	
Male/female	14 (77.8)/4 (22.2)
Child–Pugh classification, *n* (%)	
Grade A/grade B/grade C	13 (72.2)/5 (27.8)/0 (0)
Form before treatment, *n* (%)	
F1/F2/F3	6 (33.3)/7 (38.9)/5 (27.8)
Location, *n* (%)	
Ls/ Lm/ Li	0 (0)/7 (38.9)/11 (61.1)
RC sign before treatment, *n* (%)	
RC0/RC1/RC2/RC3	3 (16.7)/11 (61.1)/4 (22.2)/0 (0)
Form 1 month after treatment, *n* (%)	
F0/F1/F2/F3	4 (22.2)/11 (61.1)/3 (16.7)/0 (0)
RC sign 1 month after treatment, *n* (%)	
RC0/RC1/RC2/RC3	15 (83.3)/2 (11.1)/1 (5.6)/0(0)
Total number of punctures for all patients, times	34
Number of punctures during one session, median (SD), times	2.0 (0.90)
Technical success of EIS, *n* (%) ^†^	
Success	30 (88.2)
Failure	4 (11.8)
Clinical success of EIS, *n* (%)^†^	
Success	17 (94.4)
Failure	1 (5.6)
Adverse events related to the procedure, *n* (%)	
Fever	3 (16.6)
Chest pain	2 (11.1)
Amount of EO injected, mean (SD), mL	10 (5.79)
Amount of AS injected, mean (SD), mL	3.5 (2.74)
Effectiveness of treatment, *n* (%)	
Disappearance	5 (27.8)
Reduction	12 (66.7)
Unchanged	0 (0)
Progressive worsening	1 (5.5)
Observation period, mean (SD), day	319.5 (148.7)
Cases that required retreatment during the observation period, *n* (%)	
No/yes	13 (72.2)/5 (27.8)
Amount of gel, mean (SD), mL	265 (141)

Abbreviations: AS, 1% polidocanol (aethoxysklerol); EIS, endoscopic injection sclerotherapy; EO, 5% ethanolamine oleate mixed with a non‐ionic angiographic contrast agent; GI‐EIS, gel‐immersion endoscopic injection sclerotherapy; Li, locus inferior; Lm, locus medialis; Ls, locus superior; RC, red color; SD, standard deviation.

^†^ “Technical success of EIS” in this study was defined as puncturing the varicose vein and injecting sufficient EO to confirm the contrast of the donor vessel under fluoroscopy. “Clinical success of EIS” was defined as the reduction or disappearance of esophageal varices following the procedure.

^‡^ “Disappearance” was defined as a decrease in the varicose vein form and disappearance of high‐risk bleeding findings, such as the RC sign. “Reduction” was defined as a reduction in the varicose vein size without the complete disappearance of the RC sign. “No change” was defined as no difference after treatment. “Progressive worsening” was defined as worsening of varicose veins or RC signs after treatment.

By 1 month after treatment, five patients (27.8%) exhibited disappearance, 12 (66.7%) exhibited reduction, and one (5.5%) exhibited progressive worsening of varices, as assessed by endoscopy. The median observation duration was 319.5 days (range, 102–529 days). During this period, five patients (27.8%) had recurrent varicose veins that required retreatment, whereas the remaining 13 patients (72.2%) underwent observation without recurrence. The median amount of gel used for each GI‐EIS session was 265 mL (range, 120–600 mL).

Table 2 lists the causes of progressive worsening of varices that required retreatment. Varices were enlarged in five patients, and two of these patients experienced hepatocellular carcinoma exacerbation. The remaining three patients experienced portal vein obstruction due to portal vein thrombus, congenital portal vein deficiency, and exacerbation of cirrhosis.

**TABLE 2:** Causes of progressive worsening of varices that required retreatment.

	*N* = 5
Hepatocellular carcinoma development, *n* (%)	2 (40.0)
Portal vein obstruction due to portal vein thrombus, *n* (%)	1 (20.0)
Congenital portal vein defect, *n* (%)	1 (20.0)
Exacerbation of cirrhosis, *n* (%)	1 (20.0)

## DISCUSSION

EVL and EIS are widely performed prophylactic hemostatic procedures for esophageal varices. EVL is technically simpler than EIS, but it is associated with a higher varicose vein recurrence rate.^10^, ^11^ Consequently, EIS is often preferred for large‐volume esophageal varices with a preserved hepatic reserve. Conversely, although EIS has a low recurrence rate, the difficulty of the procedure is generally considered greater than that of EVL. In particular, during variceal puncture and sclerosing agent injection, issues such as blood vessel penetration, which causes bleeding, and improper agent injection can occur. Additionally, patients’ body movements and esophageal peristalsis may dislodge the tip of the needle. The use of gel‐immersion techniques can lower gastrointestinal tract pressure, thus reducing body movement during the procedure. Additionally, the gel, which is cooler than air, may help reduce esophageal peristalsis.^12^ With the GI‐EIS method, the gel provides a consistently clear view and enables the procedure to be performed under stable conditions. Additionally, in the event of bleeding, the bleeding point can be detected relatively easily. Therefore, this very clear view and high stability of the scope with the GI‐EIS method may improve the difficulty associated with the puncture and needle fixation.

This study revealed the effectiveness and safety of the GI‐EIS technique as well as high intravascular angiographic and clinical success rates without serious complications. Although the use of gel may increase the incidence of aspiration complications, no patients in this study experienced aspiration pneumonia. Additionally, no patients experienced respiratory conditions that worsened during the procedure. This may be due to the inflation of the endoscope with a balloon for varicose vein compression, which prevented backflow into the oral cavity. The GI‐EIS results were comparable to the conventional EIS results, suggesting that the GI‐EIS method is a safe, clinically feasible, and effective treatment option for prophylactic hemostasis of esophageal varices.^11^, ^13^ The enhanced scope stability during puncture is likely attributable to the low intra‐gastrointestinal pressure maintained by avoiding air insufflation, which allowed adequate injection of the sclerosing agent, which, in turn, may be effective for large and continuous gastric varices.

However, GI‐EIS requires more staff than the conventional EIS method because of the need for intermittent gel injection. The use of an endoscope with a water jet function that injects gel through a secondary water‐delivery tube during esophageal endoscopic submucosal dissection can reduce the risk of complications.^14^ At our hospital, we used a system that injects gel through an external irrigation tube (ERCP‐Catheter; MTW Endoskopie W Haag KG) with a water jet function during endoscopic procedures such as endoscopic hemostasis and endoscopic submucosal dissection. If additional gel was needed during the procedure, then it was added using a water‐delivery device (JW‐3 System; Fujifilm Co.) through an external irrigation tube. Conversely, a normal water supply was provided via a secondary water delivery tube. This allowed additional gel and water to be injected independently. Additionally, a foot pedal allowed the operator to add the gel; therefore, additional staff were not required. We hypothesized that this system could also be used for GI‐EIS procedures, thus addressing challenges associated with the need for additional staff.

Although we have demonstrated the feasibility of applying GI‐EIS for esophageal varices, this study had some limitations. First, this was a single‐center retrospective study that included a small number of patients. Second, the risk of varicose vein recurrence may not have been accurately assessed because of the short follow‐up period. Third, unfortunately, a detailed report of conventional EIS, including the experience of the endoscopist, number of punctures, and amount of sclerosing agent injected at the time of puncture, at our institution was not recorded. As a result, it was difficult to accurately compare the GEL‐EIS method with the conventional EIS method. Therefore, we do not yet recommend the widespread adoption of this technique for clinical use. However, we think that the effectiveness of the GI‐EIS method, with its very clear view and high scope stability, may contribute to an improved technical success rate. Further case studies and research are required to clarify these issues.

In conclusion, we demonstrated that GI‐EIS is a novel procedure that is safe, clinically feasible, and potentially effective for prophylactic hemostasis of esophageal varices.

## CONFLICT OF INTEREST STATEMENT

None.

## ETHICS STATEMENT

**Approval of the research protocol by an Institutional Reviewer Board**. The study protocol was approved by the Human Research Committee of Osaka Medical and Pharmaceutical University (Institutional Review Board no. 2024‐024) and conformed to the ethical guidelines of the 1975 Declaration of Helsinki.

## PATIENT CONSENT STATEMENT

N/A

## CLINICAL TRIAL REGISTRATION

N/A

## Supporting information

GI‐EISL_case_series_revice1.0_.mp4
